# 2-(4-Fluoro­phen­yl)-1-(3-meth­oxy­phen­yl)-4,5-dimethyl-1*H*-imidazole

**DOI:** 10.1107/S1600536813032492

**Published:** 2013-12-04

**Authors:** S. Rizwana Begum, R. Hema, N. Srinivasan, A. G. Anitha

**Affiliations:** a Department of Physics, Seethalakshmi Ramaswami College (Autonomous), Tiruchirappalli 620 002, India; b Department of Physics, K. Ramakrishnan College of Engineering, Samayapuram, Tiruchirappalli 621 112, India; c Department of Chemistry, S.K.P. Engineering College, Thiruvanamalai 606 611, India

## Abstract

In the title compound, C_18_H_17_FN_2_O, the imidazole ring makes dihedral angles of 68.81 (6) and 25.20 (8)° with the meth­oxy­phenyl and fluoro­phenyl rings, respectively. The dihedral angle between the meth­oxy­phenyl and fluoro­phenyl ring is 71.89 (6)°. In the crystal, mol­ecules are linked into inversion dimers with an *R*_2_^2^(8) graph-set motif by pairs of weak C—H⋯F inter­actions.

## Related literature   

For related structures, see: Rizwana Begum *et al.* (2013 ▶); Srinivasan *et al.* (2013 ▶); Gayathri *et al.* (2010 ▶); Rosepriya *et al.* (2011 ▶). For graph-set motifs, see: Bernstein *et al.* (1995 ▶).
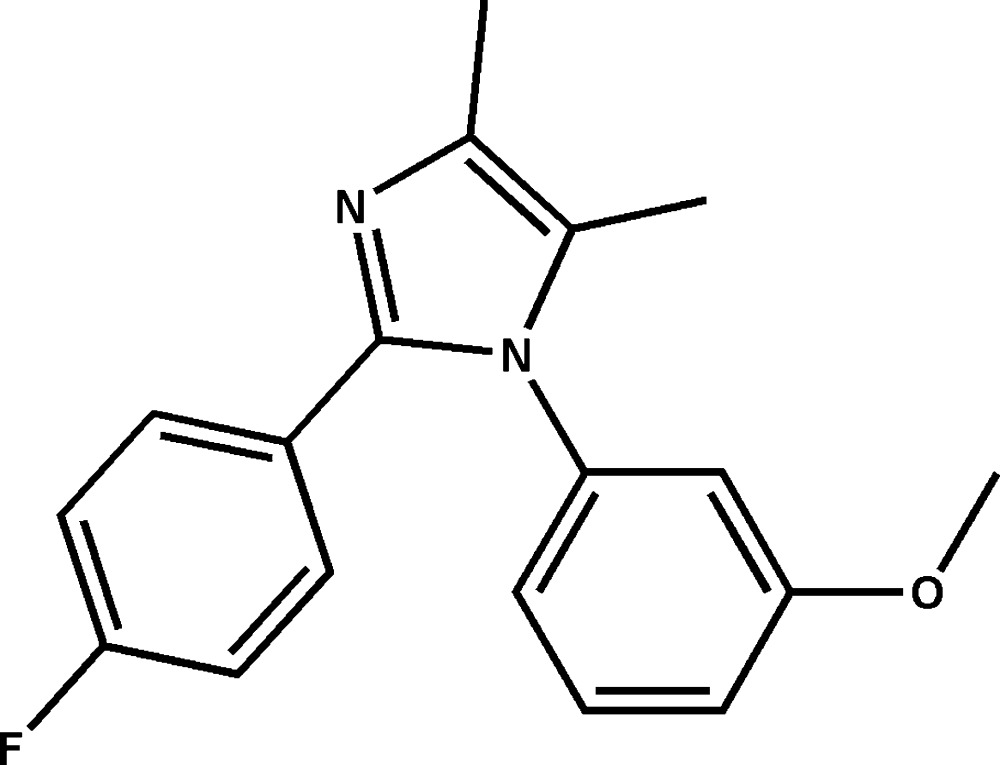


## Experimental   

### 

#### Crystal data  

C_18_H_17_FN_2_O
*M* *_r_* = 296.34
Triclinic, 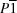

*a* = 8.1196 (5) Å
*b* = 9.6014 (6) Å
*c* = 10.6116 (6) Å
α = 106.818 (3)°
β = 92.059 (3)°
γ = 96.114 (3)°
*V* = 785.45 (8) Å^3^
*Z* = 2
Mo *K*α radiation
μ = 0.09 mm^−1^
*T* = 293 K
0.30 × 0.30 × 0.25 mm

#### Data collection  

Bruker Kappa APEXII CCD diffractometer
Absorption correction: multi-scan (*SADABS*; Bruker, 2004 ▶) *T*_min_ = 0.974, *T*_max_ = 0.979
15949 measured reflections
3597 independent reflections
2477 reflections with *I* > 2σ(*I*)
*R*_int_ = 0.031

#### Refinement  

*R*[*F* ^2^ > 2σ(*F* ^2^)] = 0.047
*wR*(*F* ^2^) = 0.148
*S* = 1.03
3597 reflections
202 parameters
H-atom parameters constrained
Δρ_max_ = 0.20 e Å^−3^
Δρ_min_ = −0.22 e Å^−3^

### 

Data collection: *APEX2* (Bruker, 2004 ▶); cell refinement: *APEX2* and *SAINT* (Bruker, 2004 ▶); data reduction: *SAINT* and *XPREP* (Bruker, 2004 ▶); program(s) used to solve structure: *SIR92* (Altomare *et al.*, 1993 ▶); program(s) used to refine structure: *SHELXL97* (Sheldrick, 2008 ▶); molecular graphics: *ORTEP-3 for Windows* (Farrugia, 2012 ▶); software used to prepare material for publication: *PLATON* (Spek, 2009 ▶).

## Supplementary Material

Crystal structure: contains datablock(s) I, global. DOI: 10.1107/S1600536813032492/hg5365sup1.cif

Structure factors: contains datablock(s) I. DOI: 10.1107/S1600536813032492/hg5365Isup2.hkl

Supporting information file. DOI: 10.1107/S1600536813032492/hg5365Isup3.cml

Additional supporting information:  crystallographic information; 3D view; checkCIF report

## Figures and Tables

**Table 1:** Hydrogen-bond geometry (Å, °)

*D*—H⋯*A*	*D*—H	H⋯*A*	*D*⋯*A*	*D*—H⋯*A*
C15—H15⋯F2^i^	0.93	2.55	3.437 (2)	160

Symmetry code: (i) 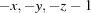.

**Fig. 1.**
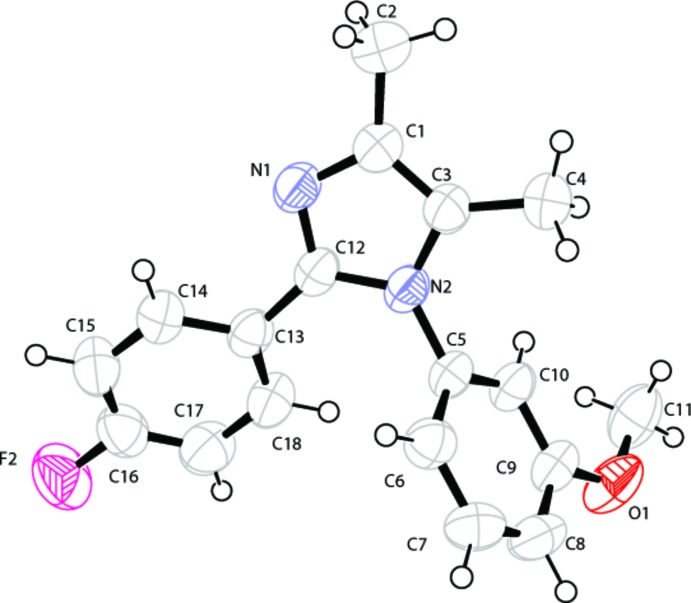
The molecular structure of the title compound,showing the atom-labelling scheme. Displacement ellipsoids are drawn at the 50% probability level. H atoms are represented by circles of arbitrary radii.

**Fig. 2.**
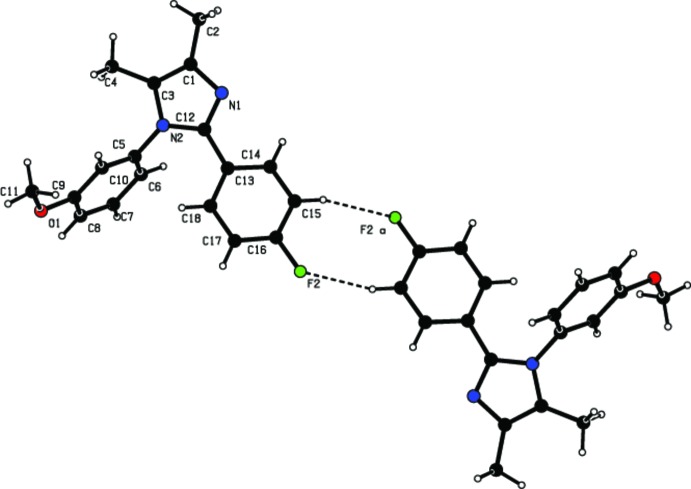
Intermolecular C-H···F hydrogen bonding in (I).

## supplementary crystallographic information

### 1. Comment

In a continuation of structural studies of 4,5-dimethyl-1*H*-imidazole derivatives (Rizwana *et al.*, 2013; Srinivasan *et al.*, 2013; Gayathri *et al.*, 2010; Rosepriya *et al.*, 2011), we have taken up the title compound, (I), for crystallographic investigation.

In (I) (Fig. 1), C_18_H_17_FN_2_O, the imidazole ring is essentially planar and makes dihedral angles of 68.81 (6)° and 25.20 (8)° with the methoxyphenyl (C5-C10) and fluorophenyl rings (C13-C18) respectively. The dihedral angle between the methoxyphenyl and fluorophenyl ring is 71.89 (6)°. The C15—H15···F2 (-*x*,-*y*,-1 - *z*) interaction link pairs of molecules across centres of inversion to form dimers with ring motif *R*_2_^2^(8) (Bernstein *et al.*, (1995)) (Fig. 2).

### 2. Experimental

To pure butane-2,3-dione (1.48 g, 15 mmol) in ethanol (10 ml), *m*-methoxy aniline (1.5 g, 15 mmol), ammonium acetate (1.15 g, 15 mmol) and 4-fluorobenzaldehyde (1.7 g, 15 mmol) was added about 1 h by maintaining the temperature at 333 K. The reaction mixture was refluxed for 7 days and extracted with dichloromethane. The solid separated was purified by column chromatography using hexane: ethyl acetate as the eluent. Yield: 1.94 g (48%).

### 3. Refinement

All H atoms were placed in geometrically idealized positions (C—H = 0.93 Å) and constrained to ride on their parent atoms with *U*_iso_(H) = 1.2*U*_eq_(C) but the methyl H atoms were located in SHELX with an ideal geometry (C—H = 0.96 Å) , *U*_iso_(H) = 1.5*U*_eq_(C).

### Crystal data

**Table:**

C_18_H_17_FN_2_O	*Z* = 2
*M**_r_* = 296.34	*F*(000) = 312
Triclinic, *P*1	*D*_x_ = 1.253 Mg m^−^^3^
Hall symbol: -P 1	Mo *K*α radiation, λ = 0.71073 Å
*a* = 8.1196 (5) Å	Cell parameters from 3597 reflections
*b* = 9.6014 (6) Å	θ = 2.5–30.3°
*c* = 10.6116 (6) Å	µ = 0.09 mm^−^^1^
α = 106.818 (3)°	*T* = 293 K
β = 92.059 (3)°	Block, colourless
γ = 96.114 (3)°	0.30 × 0.30 × 0.25 mm
*V* = 785.45 (8) Å^3^	

### Data collection

**Table:**

Bruker Kappa APEXII CCD diffractometer	3597 independent reflections
Radiation source: fine-focus sealed tube	2477 reflections with *I* > 2σ(*I*)
Graphite monochromator	*R*_int_ = 0.031
ω and φ scan	θ_max_ = 27.5°, θ_min_ = 2.2°
Absorption correction: multi-scan (*SADABS*; Bruker, 2004)	*h* = −10→10
*T*_min_ = 0.974, *T*_max_ = 0.979	*k* = −12→12
15949 measured reflections	*l* = −13→13

### Refinement

**Table:**

Refinement on *F*^2^	Primary atom site location: structure-invariant direct methods
Least-squares matrix: full	Secondary atom site location: difference Fourier map
*R*[*F*^2^ > 2σ(*F*^2^)] = 0.047	Hydrogen site location: inferred from neighbouring sites
*wR*(*F*^2^) = 0.148	H-atom parameters constrained
*S* = 1.03	*w* = 1/[σ^2^(*F*_o_^2^) + (0.0689*P*)^2^ + 0.1414*P*] where *P* = (*F*_o_^2^ + 2*F*_c_^2^)/3
3597 reflections	(Δ/σ)_max_ = 0.001
202 parameters	Δρ_max_ = 0.20 e Å^−^^3^
0 restraints	Δρ_min_ = −0.22 e Å^−^^3^

### Special details

**Table:**

Geometry. All e.s.d.'s (except the e.s.d. in the dihedral angle between two l.s. planes) are estimated using the full covariance matrix. The cell e.s.d.'s are taken into account individually in the estimation of e.s.d.'s in distances, angles and torsion angles; correlations between e.s.d.'s in cell parameters are only used when they are defined by crystal symmetry. An approximate (isotropic) treatment of cell e.s.d.'s is used for estimating e.s.d.'s involving l.s. planes.
Refinement. Refinement of *F*^2^ against ALL reflections. The weighted *R*-factor *wR* and goodness of fit *S* are based on *F*^2^, conventional *R*-factors *R* are based on *F*, with *F* set to zero for negative *F*^2^. The threshold expression of *F*^2^ > σ(*F*^2^) is used only for calculating *R*-factors(gt) *etc*. and is not relevant to the choice of reflections for refinement. *R*-factors based on *F*^2^ are statistically about twice as large as those based on *F*, and *R*-factors based on ALL data will be even larger.

### Fractional atomic coordinates and isotropic or equivalent isotropic displacement parameters (Å^2^)

**Table:**

	*x*	*y*	*z*	*U*_iso_*/*U*_eq_	
C1	0.2524 (2)	0.71718 (19)	0.06251 (16)	0.0606 (4)	
C2	0.2395 (3)	0.8699 (2)	0.0606 (2)	0.0941 (7)	
H2A	0.2741	0.9366	0.1462	0.141*	
H2B	0.1265	0.8791	0.0378	0.141*	
H2C	0.3096	0.8927	−0.0035	0.141*	
C3	0.2938 (2)	0.66931 (18)	0.16587 (15)	0.0574 (4)	
C4	0.3379 (3)	0.7478 (2)	0.30717 (17)	0.0771 (5)	
H4A	0.3221	0.8491	0.3241	0.116*	
H4B	0.4521	0.7407	0.3284	0.116*	
H4C	0.2683	0.7046	0.3605	0.116*	
C5	0.29393 (19)	0.41950 (17)	0.19342 (14)	0.0504 (4)	
C6	0.1516 (2)	0.33657 (19)	0.20825 (17)	0.0597 (4)	
H6	0.0503	0.3431	0.1679	0.072*	
C7	0.1631 (2)	0.2430 (2)	0.2848 (2)	0.0709 (5)	
H7	0.0683	0.1851	0.2952	0.085*	
C8	0.3106 (3)	0.2339 (2)	0.34526 (19)	0.0705 (5)	
H8	0.3157	0.1707	0.3968	0.085*	
C9	0.4529 (2)	0.3186 (2)	0.33012 (15)	0.0609 (4)	
C10	0.4455 (2)	0.41192 (18)	0.25281 (14)	0.0551 (4)	
H10	0.5408	0.4684	0.2410	0.066*	
C11	0.7407 (3)	0.3921 (3)	0.3874 (2)	0.0872 (6)	
H11A	0.7659	0.3754	0.2969	0.131*	
H11B	0.8308	0.3691	0.4358	0.131*	
H11C	0.7252	0.4931	0.4250	0.131*	
C12	0.23901 (18)	0.48287 (17)	−0.01663 (14)	0.0504 (4)	
C13	0.22060 (19)	0.33656 (18)	−0.11246 (14)	0.0526 (4)	
C14	0.1224 (2)	0.3145 (2)	−0.22791 (17)	0.0648 (5)	
H14	0.0651	0.3896	−0.2386	0.078*	
C15	0.1082 (3)	0.1840 (2)	−0.32662 (19)	0.0757 (5)	
H15	0.0429	0.1705	−0.4041	0.091*	
C16	0.1914 (3)	0.0748 (2)	−0.30879 (19)	0.0712 (5)	
C17	0.2885 (3)	0.0901 (2)	−0.1976 (2)	0.0758 (5)	
H17	0.3439	0.0135	−0.1880	0.091*	
C18	0.3031 (2)	0.2221 (2)	−0.09918 (17)	0.0662 (5)	
H18	0.3696	0.2344	−0.0226	0.079*	
N1	0.21906 (17)	0.60175 (15)	−0.05036 (12)	0.0574 (4)	
N2	0.28515 (16)	0.51797 (14)	0.11500 (12)	0.0509 (3)	
O1	0.59387 (18)	0.30194 (18)	0.39395 (14)	0.0866 (4)	
F2	0.17789 (18)	−0.05430 (14)	−0.40697 (13)	0.1043 (4)	

### Atomic displacement parameters (Å^2^)

**Table:**

	*U*^11^	*U*^22^	*U*^33^	*U*^12^	*U*^13^	*U*^23^
C1	0.0682 (11)	0.0603 (10)	0.0551 (9)	0.0035 (8)	0.0008 (7)	0.0217 (8)
C2	0.138 (2)	0.0661 (12)	0.0821 (14)	0.0073 (12)	−0.0133 (13)	0.0318 (11)
C3	0.0622 (10)	0.0604 (10)	0.0495 (8)	0.0004 (7)	0.0018 (7)	0.0189 (7)
C4	0.1045 (15)	0.0707 (12)	0.0521 (10)	0.0007 (10)	−0.0037 (9)	0.0166 (9)
C5	0.0560 (9)	0.0580 (9)	0.0406 (7)	0.0081 (7)	0.0066 (6)	0.0193 (7)
C6	0.0564 (9)	0.0641 (10)	0.0604 (9)	0.0043 (7)	0.0071 (7)	0.0220 (8)
C7	0.0715 (12)	0.0672 (11)	0.0807 (12)	−0.0006 (9)	0.0158 (9)	0.0342 (10)
C8	0.0898 (14)	0.0664 (11)	0.0691 (11)	0.0158 (10)	0.0175 (10)	0.0377 (9)
C9	0.0691 (11)	0.0733 (11)	0.0482 (8)	0.0194 (9)	0.0073 (7)	0.0260 (8)
C10	0.0549 (9)	0.0699 (10)	0.0460 (8)	0.0071 (7)	0.0062 (6)	0.0255 (7)
C11	0.0686 (13)	0.1295 (19)	0.0757 (13)	0.0226 (12)	−0.0032 (10)	0.0468 (13)
C12	0.0480 (8)	0.0630 (9)	0.0450 (8)	0.0069 (7)	0.0037 (6)	0.0230 (7)
C13	0.0505 (8)	0.0642 (10)	0.0463 (8)	0.0052 (7)	0.0073 (6)	0.0215 (7)
C14	0.0676 (11)	0.0681 (11)	0.0582 (10)	0.0073 (8)	−0.0056 (8)	0.0196 (8)
C15	0.0791 (13)	0.0805 (13)	0.0592 (11)	0.0014 (10)	−0.0100 (9)	0.0122 (10)
C16	0.0795 (13)	0.0648 (11)	0.0614 (11)	0.0019 (9)	0.0099 (9)	0.0081 (9)
C17	0.0929 (14)	0.0700 (12)	0.0691 (12)	0.0250 (10)	0.0141 (10)	0.0212 (10)
C18	0.0733 (11)	0.0773 (12)	0.0516 (9)	0.0212 (9)	0.0039 (8)	0.0205 (9)
N1	0.0623 (8)	0.0642 (8)	0.0497 (7)	0.0067 (6)	0.0007 (6)	0.0241 (7)
N2	0.0523 (7)	0.0595 (8)	0.0446 (6)	0.0049 (6)	0.0026 (5)	0.0222 (6)
O1	0.0781 (9)	0.1210 (12)	0.0843 (9)	0.0234 (8)	−0.0001 (7)	0.0640 (9)
F2	0.1274 (11)	0.0784 (8)	0.0861 (9)	0.0117 (7)	0.0010 (7)	−0.0073 (7)

### Geometric parameters (Å, º)

**Table:**

C1—C3	1.351 (2)	C9—C10	1.383 (2)
C1—N1	1.371 (2)	C10—H10	0.9300
C1—C2	1.488 (3)	C11—O1	1.413 (3)
C2—H2A	0.9600	C11—H11A	0.9600
C2—H2B	0.9600	C11—H11B	0.9600
C2—H2C	0.9600	C11—H11C	0.9600
C3—N2	1.389 (2)	C12—N1	1.3149 (19)
C3—C4	1.481 (2)	C12—N2	1.3667 (18)
C4—H4A	0.9600	C12—C13	1.465 (2)
C4—H4B	0.9600	C13—C18	1.384 (2)
C4—H4C	0.9600	C13—C14	1.388 (2)
C5—C6	1.372 (2)	C14—C15	1.372 (3)
C5—C10	1.381 (2)	C14—H14	0.9300
C5—N2	1.4342 (18)	C15—C16	1.360 (3)
C6—C7	1.382 (2)	C15—H15	0.9300
C6—H6	0.9300	C16—C17	1.357 (3)
C7—C8	1.361 (3)	C16—F2	1.360 (2)
C7—H7	0.9300	C17—C18	1.380 (3)
C8—C9	1.382 (3)	C17—H17	0.9300
C8—H8	0.9300	C18—H18	0.9300
C9—O1	1.357 (2)		
			
C3—C1—N1	110.58 (15)	C5—C10—H10	120.7
C3—C1—C2	128.58 (17)	C9—C10—H10	120.7
N1—C1—C2	120.83 (15)	O1—C11—H11A	109.5
C1—C2—H2A	109.5	O1—C11—H11B	109.5
C1—C2—H2B	109.5	H11A—C11—H11B	109.5
H2A—C2—H2B	109.5	O1—C11—H11C	109.5
C1—C2—H2C	109.5	H11A—C11—H11C	109.5
H2A—C2—H2C	109.5	H11B—C11—H11C	109.5
H2B—C2—H2C	109.5	N1—C12—N2	110.49 (14)
C1—C3—N2	105.43 (14)	N1—C12—C13	122.62 (13)
C1—C3—C4	131.90 (17)	N2—C12—C13	126.82 (13)
N2—C3—C4	122.66 (14)	C18—C13—C14	117.93 (16)
C3—C4—H4A	109.5	C18—C13—C12	124.26 (14)
C3—C4—H4B	109.5	C14—C13—C12	117.67 (14)
H4A—C4—H4B	109.5	C15—C14—C13	121.29 (17)
C3—C4—H4C	109.5	C15—C14—H14	119.4
H4A—C4—H4C	109.5	C13—C14—H14	119.4
H4B—C4—H4C	109.5	C16—C15—C14	118.56 (17)
C6—C5—C10	121.91 (14)	C16—C15—H15	120.7
C6—C5—N2	119.19 (14)	C14—C15—H15	120.7
C10—C5—N2	118.89 (14)	C17—C16—C15	122.62 (18)
C5—C6—C7	118.12 (16)	C17—C16—F2	118.87 (18)
C5—C6—H6	120.9	C15—C16—F2	118.51 (18)
C7—C6—H6	120.9	C16—C17—C18	118.46 (18)
C8—C7—C6	121.27 (17)	C16—C17—H17	120.8
C8—C7—H7	119.4	C18—C17—H17	120.8
C6—C7—H7	119.4	C17—C18—C13	121.13 (17)
C7—C8—C9	120.05 (16)	C17—C18—H18	119.4
C7—C8—H8	120.0	C13—C18—H18	119.4
C9—C8—H8	120.0	C12—N1—C1	106.37 (13)
O1—C9—C8	115.78 (15)	C12—N2—C3	107.12 (12)
O1—C9—C10	124.26 (16)	C12—N2—C5	127.45 (13)
C8—C9—C10	119.96 (16)	C3—N2—C5	124.53 (12)
C5—C10—C9	118.68 (15)	C9—O1—C11	117.83 (14)
			
N1—C1—C3—N2	−0.23 (19)	C15—C16—C17—C18	−0.1 (3)
C2—C1—C3—N2	179.2 (2)	F2—C16—C17—C18	179.23 (17)
N1—C1—C3—C4	−179.06 (18)	C16—C17—C18—C13	0.3 (3)
C2—C1—C3—C4	0.4 (4)	C14—C13—C18—C17	0.1 (3)
C10—C5—C6—C7	−0.3 (2)	C12—C13—C18—C17	−175.54 (16)
N2—C5—C6—C7	−179.69 (15)	N2—C12—N1—C1	−0.27 (17)
C5—C6—C7—C8	0.7 (3)	C13—C12—N1—C1	−177.54 (14)
C6—C7—C8—C9	−0.4 (3)	C3—C1—N1—C12	0.32 (19)
C7—C8—C9—O1	−179.92 (17)	C2—C1—N1—C12	−179.21 (18)
C7—C8—C9—C10	−0.4 (3)	N1—C12—N2—C3	0.13 (17)
C6—C5—C10—C9	−0.5 (2)	C13—C12—N2—C3	177.25 (14)
N2—C5—C10—C9	178.94 (14)	N1—C12—N2—C5	169.55 (14)
O1—C9—C10—C5	−179.70 (15)	C13—C12—N2—C5	−13.3 (2)
C8—C9—C10—C5	0.8 (2)	C1—C3—N2—C12	0.07 (17)
N1—C12—C13—C18	151.38 (16)	C4—C3—N2—C12	179.02 (16)
N2—C12—C13—C18	−25.4 (2)	C1—C3—N2—C5	−169.74 (15)
N1—C12—C13—C14	−24.2 (2)	C4—C3—N2—C5	9.2 (2)
N2—C12—C13—C14	158.97 (15)	C6—C5—N2—C12	−62.4 (2)
C18—C13—C14—C15	−0.5 (3)	C10—C5—N2—C12	118.14 (17)
C12—C13—C14—C15	175.38 (16)	C6—C5—N2—C3	105.29 (18)
C13—C14—C15—C16	0.7 (3)	C10—C5—N2—C3	−74.2 (2)
C14—C15—C16—C17	−0.3 (3)	C8—C9—O1—C11	−176.56 (17)
C14—C15—C16—F2	−179.68 (17)	C10—C9—O1—C11	3.9 (3)

### Hydrogen-bond geometry (Å, º)

**Table:**

*D*—H···*A*	*D*—H	H···*A*	*D*···*A*	*D*—H···*A*
C15—H15···F2^i^	0.93	2.55	3.437 (2)	160

Symmetry code: (i) −*x*, −*y*, −*z*−1.
